# Photizo: an open-source library for cross-sample analysis of FTIR spectroscopy data

**DOI:** 10.1093/bioinformatics/btac346

**Published:** 2022-05-24

**Authors:** Melissa Grant-Peters, Charlotte Rich-Griffin, Jonathan E Grant-Peters, Gianfelice Cinque, Calliope A Dendrou

**Affiliations:** Nuffield Department of Medicine, Wellcome Centre for Human Genetics, University of Oxford, Oxford OX3 7BN, UK; Nuffield Department of Medicine, Wellcome Centre for Human Genetics, University of Oxford, Oxford OX3 7BN, UK; Mathematical Institute, University of Oxford, Oxford OX2 6GG, UK; Diamond Light Source, Didcot OX11 0DE, UK; Nuffield Department of Medicine, Wellcome Centre for Human Genetics, University of Oxford, Oxford OX3 7BN, UK

## Abstract

**Motivation:**

With continually improved instrumentation, Fourier transform infrared (FTIR) microspectroscopy can now be used to capture thousands of high-resolution spectra for chemical characterization of a sample. The spatially resolved nature of this method lends itself well to histological profiling of complex biological specimens. However, current software can make joint analysis of multiple samples challenging and, for large datasets, computationally infeasible.

**Results:**

To overcome these limitations, we have developed Photizo—an open-source Python library enabling high-throughput spectral data pre-processing, visualization and downstream analysis, including principal component analysis, clustering, macromolecular quantification and mapping. Photizo can be used for analysis of data without a spatial component, as well as spatially resolved data, obtained e.g. by scanning mode IR microspectroscopy and IR imaging by focal plane array detector.

**Availability and implementation:**

The code underlying this article is available at https://github.com/DendrouLab/Photizo with access to example data available at https://zenodo.org/record/6417982#.Yk2O9TfMI6A.

## 1 Introduction

Fourier transform infrared microspectroscopy (µFTIR) enables non-destructive and label-free mapping of complex chemical information. The spatially resolved nature of this method lends itself well to the analysis of architecturally complex samples such as those of biological nature (Baker *et al.*, 2014). The functional group specificity of µFTIR provides insight into biological queries, capturing relevant molecules such as lipids, proteins, nucleic acids and carbohydrates (Bellisola and Sorio, 2012).

Continually improving instrumentation and spectral analysis methods are increasing the applicability of vibrational spectroscopy methods for disease characterization and diagnosis. These methods have repeatedly been shown to partition data based on these spectral features, distinguishing biochemical profiles of healthy control samples from pathological specimens (Heraud *et al.*, 2010; Kneipp *et al.*, 2000; Martel *et al.*, 2020). In the context of histological characterization with µFTIR specifically, clustering has been leveraged to distinguish the biochemical profile of different degrees of pathology within a given sample, with performance being comparable to a trained pathologist (Wehbe *et al.*, 2015).

Carrying out this type of analysis across multiple samples is challenging with currently available software. Commercially available options—while rich in analysis functionality—can be computationally costly to run, often limiting processing to one sample at a time. When samples are processed and analyzed individually with long running times for each step, this can increase errors and be less systematic, thereby potentially compromising reproducibility. Quasar, a recently available open-source spectroscopic data analysis toolbox extending the Orange suite, has overcome some of these challenges (Toplak *et al.*, 2021). However, its interactive interface comes at the cost of the capacity for cluster computing—a necessity for analysis of large multi-sample datasets in a timely fashion.

With multi-modal analysis approaches gaining prominence in the life and medical sciences to aid biological discovery and provide insights for patient prognosis, diagnosis and therapy (Eddy *et al.*, 2020; Miao *et al.*, 2021; Palla *et al.*, 2022), an open-source tool for streamlined analysis of µFTIR data could unlock the significant potential of this method to better characterize the relatively understudied biochemical profile of tissues, and the data generated could then be integrated with other data modalities. This would substantially increase the utility of µFTIR beyond data partitioning, and enable its use in disease characterization since multi-modal and integrative approaches enable streamlined data exploration and validation linking specific cellular processes to key macromolecular features.

**Fig. 1.**
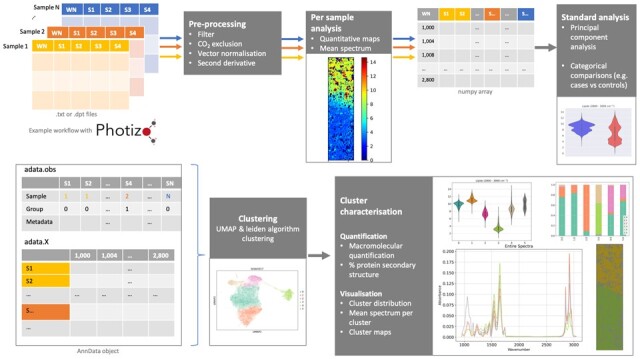
Example workflow of µFTIR data in Photizo. The Photizo workflow includes pre-processing, PCA, clustering and cluster quantification and visualization solutions for FTIR spectroscopy and imaging data. WN, wavenumber

In order to address this need, we present Photizo—an open-source Python library which makes use of the SCANPY library (Wolf *et al.*, 2018) and AnnData objects to enable spectral analysis while preserving spectra-level clinical data annotation. It includes pre-processing, analysis and visualization functions, including spatial mapping of spectra (Fig. 1).

## 2 Materials and methods

### 2.1 Inputs and pre-processing

Data inputted into Photizo are read into a numpy array for pre-processing steps. Following pre-processing of each sample, subsequent steps can be performed for individual samples or for joint analysis of multiple samples. If using a data frame with multiple samples, we recommend creating an annotation data frame in pandas containing sample information (e.g. sample name, clinical data). This is necessary for visualization of clinical variables and of single-sample data.

Photizo pre-processing allows exclusion of outlier spectra with evidence of light scattering and spectra in regions with signal indicative of no sample (e.g. sample holes, regions outside of sample borders), enabling application of vector normalization to only spectra of interest. Positions of excluded spectra are saved for repopulation prior to spatial mapping. We recommend spatially verifying the position of excluded spectra to ensure consistency with histological features (e.g. holes).

Pre-processing also enables the exclusion of the CO_2_ region, which is useful when the CO_2_ captured is of atmospheric origin and does not contribute to the analysis. Excluding this region prior to clustering ensures that atmospheric alterations do not create batch effects. Calculating the second derivative of the spectra is also included in Photizo, which controls for baseline variation at the time of collection, thereby also minimizing batch effects in subsequent clustering.

### 2.2 Analysis

#### 2.2.1 Principal component analysis

Principal component analysis (PCA) can be used as a dimensionality reduction method and can be useful for identification of batch or spectral baseline effects prior to further analyses, and for discovering variables of genuine interest. Photizo has a PCA function optimized for spectral data, that rapidly generates cumulative explained variance plots and a plot of the top eigen-spectra. The PCA outputs can also be used for principal component projection and custom plotting.

#### 2.2.2 Clustering

Photizo includes clustering tools which make use of uniform manifold approximation and projection dimensionality reduction (Becht *et al.*, 2019) paired with the Leiden algorithm (Traag *et al.*, 2019) for community detection. Clustering may be performed with entire spectra or with a particular region of interest using the region selection functions.

#### 2.2.3 Visualization and quantification

Cluster profiling benefits from functions for visual spectral inspection. Tools for quantitative comparisons also contribute to cluster characterization, with functions implemented for numerical integration of the area below the spectra within the wavenumber window of interest. Selection of the window of interest may be verified with a specific spectral inspection function, whereby the user can account for subtle peak shifts in the data to select integration windows consistent with the collected data. Resulting quantified values can be used for statistical comparisons and visualized using violin plots.

Among the quantitative measures generated as outputs are estimates for secondary structure composition derived from the spectral features; these do not rely on spectral decomposition, but rather use statistically estimated content previously reported in the literature (Goormaghtigh *et al.*, 2009), making this approach robust and reproducible.

Two key visualization functions in Photizo enable spatial mapping of data in the configuration of data collection, requiring only the number of spectra obtained in the *x* and *y* axes at time of collection. The first function maps integrated values for visualization of chemical content estimation across the tissue for a particular region of interest. The second enables spatial mapping of cluster classification. This feature is key for comparison with histological characterization and permits correlative analysis or integration (using machine learning-, topological- or tensor-based approaches, e.g.) with other spatially resolved molecular profiling methods applied to adjacent tissue sections, such as spatial transcriptomics, imaging mass spectrometry or spatial proteomics.

### 2.3 Example workflow and reference dataset

To facilitate the use of the library by new users, we have made available infrared imaging by focal plane array detector data, with spatially resolved spectra collected from brain sections for exploration of the library’s functionality. This includes areas from three neurodegenerative disease cases and three controls, enabling performance of a full workflow with reference figures, data and metadata prior to using the library on their own data.

## 3 Conclusions

Here, we present Photizo, an open-source library for analysis of FTIR spectroscopy data, which includes functionality for analyzing spatially resolved µFTIR data. This library is built in Python—a popular programming language with noted code readability—enabling users to analyze FTIR data with more flexibility regarding sample number and data size than currently available options, all at a low monetary cost. Photizo streamlines analysis of multiple samples, including the option of joint sample analysis, making its methods reproducible and easy to standardize across samples and datasets. Being built on Python, it can also be used for scripts submitted to cluster computing, vastly reducing computational costs for analysis. It has flexible functionality, facilitating reusability of basic functions and can be easily integrated into further workflows or analyses (e.g. statistical comparison of quantitative findings), and may also be adapted to the analysis of other vibrational spectroscopy methods. Importantly, while certain tools utilized for Photizo come from biomedical sciences, the library is specimen-agnostic and can easily be used for spectral analysis of other sample types.

With the rise of integrative multi-modal analysis, this package contributes to closing the gap for µFTIR data to be analyzed as part of larger integrative studies, providing biochemical context for other omics technologies. Jointly, these features contribute to maximizing the utility of spectroscopy data at lower costs, increased options for automation and streamlined but flexible processing of large datasets.

## Funding

This work was performed with support from the Wellcome Trust and Royal Society (204290/Z/16/Z) to CAD, and the Interdisciplinary Bioscience DTP, supported by the BBSRC to MG-P. Tissue samples and associated clinical and neuropathological data were supplied by the Multiple Sclerosis Society Tissue Bank, funded by the Multiple Sclerosis Society of Great Britain and Northern Ireland, registered charity 207495.

*Conflict of Interest*: none declared.

## References

[btac346-B1] Baker M.J. et al (2014) Using Fourier transform IR spectroscopy to analyze biological materials. Nat. Protoc., 9, 1771–1791. 24992094 10.1038/nprot.2014.110 PMC4480339

[btac346-B2] Becht E. et al (2019) Dimensionality reduction for visualizing single-cell data using UMAP. Nat. Biotechnol., 37, 38–44. 10.1038/nbt.4314 30531897

[btac346-B3] Bellisola G. , Sorio C. (2012) Infrared spectroscopy and microscopy in cancer research and diagnosis. Am. J. Cancer Res., 2, 1–21. 22206042 PMC3236568

[btac346-B4] Eddy S. et al (2020) Integrated multi-omics approaches to improve classification of chronic kidney disease. Nat. Rev. Nephrol., 16, 657–668. 32424281 10.1038/s41581-020-0286-5

[btac346-B5] Goormaghtigh E. et al (2009) Protein secondary structure content in solution, films and tissues: redundancy and complementarity of the information content in circular dichroism, transmission and ATR FTIR spectra. Biochim. Biophys. Acta, 1794, 1332–1343. 19540367 10.1016/j.bbapap.2009.06.007

[btac346-B6] Heraud P. et al (2010) Early detection of the chemical changes occurring during the induction and prevention of autoimmune-mediated demyelination detected by FT-IR imaging. Neuroimage, 49, 1180–1189. 19796690 10.1016/j.neuroimage.2009.09.053

[btac346-B7] Kneipp J. et al (2000) Detection of pathological molecular alterations in scrapie-infected hamster brain by Fourier transform infrared (FT-IR) spectroscopy. Biochim. Biophys. Acta, 1501, 189–199. 10838192 10.1016/s0925-4439(00)00021-1

[btac346-B8] Martel C. et al (2020) Diagnosis of idiopathic amyotrophic lateral sclerosis using Fourier-transform infrared spectroscopic analysis of patient-derived skin. Analyst, 145, 3678–3685. 32307493 10.1039/c9an02282a

[btac346-B9] Miao Z. et al (2021) Multi-omics integration in the age of million single-cell data. Nat. Rev. Nephrol., 17, 710–724. 34417589 10.1038/s41581-021-00463-x PMC9191639

[btac346-B10] Palla G. et al (2022) Spatial components of molecular tissue biology. Nat. Biotechnol., 40, 308–318. 35132261 10.1038/s41587-021-01182-1

[btac346-B11] Toplak M. et al (2021) Quasar: easy machine learning for biospectroscopy. Cells, 10, 2300. 34571947 10.3390/cells10092300 PMC8466383

[btac346-B12] Traag V.A. et al (2019) From Louvain to Leiden: guaranteeing well-connected communities. Sci. Rep., 9, 5233. 30914743 10.1038/s41598-019-41695-z PMC6435756

[btac346-B13] Wehbe K. et al (2015) Discrimination between two different grades of human glioma based on blood vessel infrared spectral imaging. Anal. Bioanal. Chem., 407, 7295–7305. 26168973 10.1007/s00216-015-8891-z PMC4569654

[btac346-B14] Wolf F.A. et al (2018) SCANPY: large-scale single-cell gene expression data analysis. Genome Biol., 19, 15. 29409532 10.1186/s13059-017-1382-0 PMC5802054

